# Multiomics kaleidoscope to visualize cancer hallmarks

**DOI:** 10.1186/s13059-020-02176-z

**Published:** 2020-10-15

**Authors:** Shengtao Zhou

**Affiliations:** Department of Obstetrics and Gynecology, Key Laboratory of Birth Defects and Related Diseases of Women and Children of MOE and State Key Laboratory of Biotherapy, West China Second Hospital, Sichuan University and Collaborative Innovation Center, Chengdu, 610041 People’s Republic of China

Today, we have entered a data-explosive realm, which requires us to have a rational and clear viewpoint to visualize the underlying contour of a spectrum of events, especially life sciences. Cancer biology, as an important branch of life sciences, also experiences an explosion of data and related molecular characterization. Our evolving understanding of cancer hallmarks derives from rapid progress in the development of multiomics technologies. According to PubMed, omics-based investigations, either single omics or multiomics, of a variety of cancer types have been expanding each year. Correspondingly, our conception of this deadly disease no longer stays at a mono-gene level, but becomes more multidimensional and network-based. In this context, *Genome Biology* has launched this special issue entitled “Cancer Evolution and Metastasis” incorporating articles that give us additional explanation for the molecular mechanisms driving cancer evolution, heterogeneity, and metastasis. These resources will give us a bird’s-eye view of how intra-tumor and inter-tumor heterogeneity formed and how we can rationally design optimized treatment strategies based upon these discoveries.

## The central dogma-based profiling

The central dogma for molecular inheritance states that DNA makes RNA, which makes protein. This anatomy of the living phenomenon has led to subsequent development of a series of omics-based technologies, for instance, genome-wide association studies (GWAS), transcriptome-wide association studies (TWAS), and proteome-wide association studies (PWAS). Nearly a decade ago, GWAS-based sequencing technologies have been prevalent to identify associations between genetic variations and phenotypic traits, including cancers. However, while indeed thousands of novel cancer susceptibility loci have been unraveled, seldom have been translated into clinical use or show any direct biological relevance to tumorigenesis [1]. With these controversies, much attention has turned to transcriptome-level (transcriptomics) and proteome-level (proteomics) studies, especially when the scientific community calls now “a post-genomics era.” Indeed, global mapping of transcriptome or proteome profiles, which are direct effectors of the living information, more straightly links cancer phenotypes with molecular mechanisms. Furthermore, TWAS integrates GWAS and gene expression datasets to identify gene–trait associations, which could accurately prioritize the likely causal gene as well as loci where TWAS prioritizes multiple genes, some likely to be non-causal, due to sharing of expression quantitative trait loci (eQTL) [2]. More recently, the state-of-the-art PWAS, which aggregates the signal of all variants jointly impacting on a protein-coding gene and evaluates their overall effects on the protein’s function using mathematical models, has entered the stage. It could assess whether the gene exhibits functional variability between individuals that correlates with the phenotype of interest, including tumors [3]. We could anticipate more of these central dogma-based omics strategies to help us better understand cancer.

## Omics that go beyond the central dogma

Apart from the DNA-RNA-protein principle, technical advancements in chemistry and physics have dramatically deepened our conception of the living world beyond the central dogma itself. We gradually got to know that modifications of DNA (epigenomics), RNA (epitranscriptomics), and proteins (post-translational modifications), respectively, could also dramatically impact on different phenotypes. Although with insufficient evidence, it was still reported that these modifications on different levels could “trans-dimensionally” affect each other. In particular, during the last decade, a series of new forms of post-translational modifications (PTMs) have been identified, apart from the conventional protein phosphorylation, acetylation, and ubiquitination. These new types of protein PTMs, either histone proteins or non-histone proteins, include lysine butyrylation, crotonylation, succinylation, malonylation, glutarylation, and the latest discovered lactylation [4]. They have been demonstrated to serve a wide spectrum of functions under both pathological and physiological circumstances. Large-scale new protein PTM characterization, combined with other omics investigations, in different cancer phenotypes and cancer types is currently undergoing globally.

Other omics investigations that go beyond the RNA-DNA-protein principle, while not touched upon in this special issue, include metabolomics and metagenomics. Nevertheless, without proper integration strategies, all these omics investigations could only be exploited on merely a single dimension, which is inevitably lopsided.

## Integration of multiomics data in the exploration of cancer biology and evolution

To grasp an integral and comprehensive landscape of a specific phenotype with multiple-level omics data available, computational biologists have designed a spectrum of pipelines or algorithms to enable integration of data across different multiomics layers. The primary strategies for multiomics data integration could be categorized into the genome first approach, the phenotype first approach, and the environment first approach. The most frequently applied integration approaches involve simple correlation or co-mapping [5]. Two renowned projects using multiomics to study cancer on a large scale include The Cancer Genome Atlas Program (TCGA) and Clinical Proteomic Tumor Analysis Consortium (CPTAC). Groups of biologists, computational biologists, and clinicians collaborated to characterize a variety of cancer types on multidimensional levels including genomics, transcriptomics, proteomics, and epigenomics. These gigantic amounts of data not only provide insights into disease pathogenesis, but also offer extremely useful information on molecular subtyping, prognosis prediction, and drug target identification. Underlying unknowns are still waiting to be unraveled by future in-depth mining of those data.

Previously, the origins and evolutionary history of cancer are hard to be tracked. With these multiomics techniques combined with emerging evolutionary analytical tools, it becomes increasingly achievable. It would always be fascinating to map the clonal evolution of a specific tumor or trace the evolutionary tree of a tumor at different sites within one human body. A large program entitled “TRACERx Consortium” is still ongoing, which is a large-scale genomic study to advance the most current model of cancer. It views tumors not as a single entity, but as an ecosystem and a dynamic population that evolve and adapt to different cues. This unique perspective might help us pinpoint cancer vulnerabilities from an evolutionary perspective and inevitability lead to discovery of novel cancer therapeutic strategies.

## Where do future cancer multiomics go?

Our conception of the world always evolves from “macro” to “micro.” Initial attempts to understand cancer biology have been staying on the bulk level for a long time until the emergence of single-cell transcriptomics technologies. These novel techniques facilitated the community to visualize tumors at a single-cell resolution. As the approaches for proteomics and epigenetics profiling have so dramatically advanced that even a scare amount of proteins or DNAs are sufficient for characterization, single-cell proteomics, single-cell epigenomics, and even single-cell metabolomics strategies have become feasible. This paradigm shift of modern technologies facilitates simultaneous profiling of single tumor cells on a multidimensional scale. In the future, single-cell multiomics profiling would be easily accessible to the scientific community, helping grasp a more comprehensive and microscopic landscape of cancer cells. Moreover, as cancer is a complex entity and cancer cells are always surrounded by a dynamic and complicated neighborhood (tumor microenvironment), spatiotemporal information should be taken into consideration when we are conducting omics-based profiling. Integrated spatiotemporal multiomics strategies will soon emerge to give us a stereoscopic landscape of cancer and offer new clues on the development of more precise targeted therapy regimens for cancer patients.
